# The evaluation of oxidative stress and inflammation markers in serum and saliva of the patients with temporomandibular disorders

**DOI:** 10.55730/1300-0144.5737

**Published:** 2023-11-24

**Authors:** Dilara KAZAN, Burcu BAŞ AKKOR, Abdurrahman AKSOY, Enes ATMACA

**Affiliations:** 1 Department of Oral and Maxillofacial Surgery, Faculty of Dentistry, Bahçeşehir University, İstanbul, Turkiye; 2 Department of Oral and Maxillofacial Surgery, Faculty of Dentistry, Ondokuz Mayıs University, Samsun, Turkiye; 3 Department of Pharmacology and Toxicology, Faculty of Veterinary Medicine, Ondokuz Mayıs University, Samsun, Turkiye

**Keywords:** Temporomandibular disorders, oxidative stress, inflammation

## Abstract

**Background/aim:**

Temporomandibular Disorders (TMD), as in the occurrence of many diseases, have been associated with oxidative stress (OS) resulting from the disruption of antioxidant mechanisms and the accumulation of reactive oxygen species in tissues. This study was designed to compare salivary and serum OS and inflammation markers of individuals with TMD and healthy subjects.

**Materials and methods:**

A prospective cross-sectional study was conducted. Twenty-seven TMD patients diagnosed with disc displacement (DD) according to Research Diagnostic Criteria for Temporomandibular Disorders (RDC/TMD) and 17 healthy subjects were enrolled in the study. Prior to any treatment, serum, and saliva samples were taken from the patients and centrifuged, and stored at −80 °C until analyzed. All samples were examined for Interleukin-6 (IL-6), Malondialdehyde (MDA), and 8-hydroxydeoxyguanosine (8-OHdG) concentrations.

**Results:**

There was no significant difference between the groups regarding median values of 8-OHdG, IL-6, and MDA (p > 0.05). When the relationship between serum and salivary 8-OHdG, IL-6, and MDA levels in all subjects was evaluated, there was a strong positive correlation between the levels of 8-OHdG and IL-6 in the serum (r = 0.752, p <0.001). In the study group, when the relationship between pain levels and serum and saliva 8-OHdG, IL-6, and MDA levels was assessed, a positive and strong correlation was found between the levels of 8-OHdG and IL-6 in serum.

**Conclusion:**

Although the strong correlation between pain scores and serum 8-OHdG and MDA levels supports the hypothesis that inflammation and OS mechanisms may be interrelated, according to the results of the study, inflammatory and OS markers in patients with TMD were not different from healthy individuals.

## 1. Introduction

Temporomandibular disorders (TMD) are a group of diseases characterized by symptoms such as pain in the joint area, clicking or crepitation sound and limitation of mouth opening. Research has suggested that the most important factors underlying TMD are oxidative stress (OS) and inflammation resulting from direct or indirect trauma to the Temporomandibular Joint (TMJ) region [1].

The term OS is defined as any condition that causes free radical accumulation in tissue. OS, which is an indicator of the deterioration of the balance between oxidants/antioxidants in an organism, is thought to play a role in the pathogenesis of various diseases such as atherosclerosis, aging, cancer, neurological disorders, diabetes, ischemia/reperfusion, Alzheimer’s, Parkinson’s, rheumatoid arthritis and chronic inflammation [2,3]. Recently, many studies have shown a relationship between the pathogenesis of TMD and various free radicals, antioxidant enzymes, and inflammation mediators. Through biomolecule methods, studies determine the increase of some cytokines such as Interleukin 1β (IL-1β), Interleukin-6 (IL-6) in TMJ with internal derangement [4]. These studies emphasize the positive relationship between the severity of the disease and the levels of synovial fluid mediators in joints with TMD. Although it has been supported by previous studies that synovial fluid is a reliable diagnostic material that reflects the pathological condition in the joint, the disadvantages of the difficulty of obtaining have led to the need for the use of another reliable diagnostic tool. Saliva and blood are materials that are easily available and provide information about the formation and etiology of many systemic diseases.

There are studies in the literature examining OS and inflammation markers in saliva and blood in TMD patients. An association between higher levels of OS markers such as with higher pain intensity scores has been reported before [5–8]. In studies, mostly patients with painful TMD were compared with healthy individuals. Malondialdehyde (MDA), which is the last product of the lipid peroxidation reaction generated by free radicals and, 8-hydroxydeoxyguanosine (8-OHdG), a direct indicator of DNA destruction, have been reported to be at high concentrations in patients with inflammation or acute pain [5].

However, TMD is a broad name of disorder that has different pathogenesis and clinical presentation. In order to reach more reliable scientific results in TMD studies, more specific subgroups should be determined with evidence-based diagnostic tools in studies. Research Diagnostic Criteria for TMD (RDC/TMD) is a two-axis system that diagnoses and classifies within the biopsychosocial model and increases the level of consistency of studies through the use of standardized diagnostic criteria [9,10].

The aim of this study was to compare the salivary and serum OS and inflammatory marker levels of patients diagnosed with disc displacement (DD) with healthy subjects. For this purpose, IL-6, MDA, and 8-OHdG were evaluated as biomarkers of inflammation and OS.

## 2. Materials and methods

### 2.1. Study design and participants

This prospective study was carried out in the Oral and Maxillofacial Surgery Department of Ondokuz Mayıs University, Faculty of Dentistry after approval from the local institutional review board (Clinical Research Ethics Committee of Ondokuz Mayıs University Experimental Medicine Research and Application Centre-2017/245). All procedures performed involving human participants were conducted in accordance with the Declaration of Helsinki. Informed consent was obtained from all participants included in the study.

The study group was composed of patients who applied to our clinic with complaints of pain in TMJ region. Following clinical examination, patients with TMD were diagnosed using the tools of RDC/TMD [10]. Power analysis was set based on the study of Lawaf et al. [11]. 27 TMD subjects who had RDC/TMD type II disorder (TMJ-DD with reduction, TMJ-DD without reduction with limited opening, TMJ-DD without reduction without limited opening) and, 22 age and sex matched volunteers were included as the control group. In the OS analysis, 5 patients were dropped since saliva samples were insufficient to perform analysis. Ultimately, 17 control samples had all of the required measurements.

The participants were asked about general health conditions, medication intake, and smoking addiction. Individuals that have other local or systemic diseases, pain of dental origin, pregnancy or lactation, story of the use of antiinflammatory drugs, analgesics, muscle relaxants, vitamin C or vitamin E, smoking, and individuals who had already been under treatment for TMD were excluded from the study.

### 2.2. Clinical examination

A single examiner, a specialist in TMD performed the clinical examination. All participants were evaluated by means of a questionnaire of RDC/TMD. The patients completed a visual analogue scale (VAS) to assess their pain, with marked endpoints of score 0 (no pain) and score 10 (worst pain ever experienced).

### 2.3. Saliva collection

Subjects were asked to restrain from using lipstick, chewing gum, eating, or drinking any liquids except water for two h before sampling saliva. They rinsed their mouth for 30 s with clean water and the water was discarded. Subjects were instructed not to try to generate saliva, talk, or think about food. Unstimulated saliva was collected for 5 min into a graduated sterile tube. After centrifugation at 3000 rpm for 10 min, saliva samples were stored at −80 °C until analyzed. The sample collection and storage principles used in the study are described in detail by Vrbanovic et al. [12].

### 2.4. Serum collection

Venous blood (8.5 mL) was collected in Serum Tubes (BD Vacutainer® Plus Serum Tubes). Tubes were inverted 10 times and allowed to clot for 30 min. Serum was harvested after centrifugation at 3000 rpm for 10 min. Collected serum samples were stored at −80 °C until analyzed.

### 2.5. Biochemical analysis

All the samples were analyzed to determine the concentrations of IL-6, MDA, and 8-OHdG. The levels of 8-OHdG and IL-6 in both saliva and serum were determined using the Enzyme Linked Immuno Sorbent Assay (ELISA) method, following the instructions provided by the kit manufacturer. The levels of MDA in both saliva and serum were determined using the High Performance Liquid Chromatography with Fluorescent Detector (HPLC-FLD) method, as specified by Yoshioka et al. [13], with the device settings adjusted in accordance with the method described by Agarwal and Chase [14].

### 2.6. Statistical analysis

Analysis of the data obtained was performed using SPSS 12.0, SPSS Inc., Chicago, Illinois, USA. Descriptive statistics (mean, standard deviation, minimum, maximum, median) were used to describe continuous variables. The normal distribution of the data was evaluated with the “Saphiro-Wilk” test. The difference between control and study group was compared using the Mann Whitney U test. The statistical significance level was determined as 0.05 at a 95% confidence interval. Spearman correlation analysis was used to evaluate the relationship between parameters and between pain and parameters.

## 3. Results

This study was carried out on a total of 44 patients (30 males and 14 females) aged between 14 and 40 years. The descriptive data of all participants are summarized in Table 1. There was no statistically significant difference between the study and control groups in terms of mean age. No statistically significant difference was found between serum and saliva 8-OHdG, IL-6- and MDA levels of male and female subjects (Table 2).

There was no difference between the study group and the control group in terms of serum and saliva 8-OHdG, IL-6, and MDA median values (p > 0.05) (Table 3).

When the relationship between pain values and serum and salivary 8-OHdG, IL-6, and MDA levels in the study group was evaluated, a strong positive correlation was found between pain and serum 8-OHdG and IL-6 levels (Table 4).

When the relationship between serum and saliva 8-OHdG, IL-6, and MDA levels in all samples in both groups were evaluated, a strong positive correlation was found between serum 8-OHdG and IL-6 levels (r = 0.752; p < 0.001).

## 4. Discussion

TMD involve a clinically progressive process that begins with changes at the molecular level and can result in hard tissue destruction. In recent years, studies have focused on the disruption of the balance between oxidant and antioxidants in the pathogenesis of TMD [8]. It is thought that inflammation in TMJ is associated with OS due to the accumulation of free radicals, and tissue damage occurs as an inflammatory response [15,16]. The expression OS is adopted to describe any condition that results in an accumulation of free radicals which are deactivated by molecules known as antioxidants. It is still controversial whether these products are the cause of TMD or the result of the existing pathology [17]. It is possible that mechanical stress in the TMJ and masticatory muscles can produce free radicals through a number of mechanisms that exacerbate tissue damage, inflammation, and pain [18]. Free radical accumulation in the joint may occur with mechanical tension suppressing the local antioxidant defense, internal irregularities, and damage to the joint tissues [18–20]. It is known that hypoxia damage caused by repeated strain on the muscles can increase the formation of free radicals and pain inflammatory mediators [21].

In recent years, blood and saliva, as well as TMJ synovial fluid, have been used to detect free radicals and degeneration markers in TMD patients [22–31]. Saliva is the protective fluid of the oral cavity and has been suggested as a potential diagnostic tool for many conditions [5,6,32]. Unlike blood and synovial fluid, salivary tests are less invasive and can be performed without special equipment or personnel. Consisting of both enzymatic and nonenzymatic antioxidants, saliva plays a critical role as part of the antioxidant system [6,7]. Many degenerative diseases are systemic and manifest with an increase in inflammatory cells and mediators in the complete blood count. To date, several studies have shown the relationship between TMD and blood and salivary markers of inflammation. However, more studies are needed to answer the question of whether these markers can be specific markers of TMD. For this purpose, we aimed to determine whether the salivary and serum OS and inflammation mediators of TMD patients are different than in healthy individuals. As free radical markers we used 8-OHdG, which gives sensitive results because it is a direct sign of DNA destruction, and MDA, which is the most widely used mediator in the literature in the examination of free radicals. Studies have shown high levels of both 8-OHdG and MDA associated with higher pain intensity scores in TMD patients [5]. As an inflammatory mediator, we preferred IL-6, one of the most important proinflammatory cytokines, which has been used to show the relationship between inflammatory processes and the destruction of TMJ components in previous studies.

Sotillo et al. [5] compared saliva and serum samples of 20 painful TMD patients and 10 healthy controls in their pilot study. Patients who have type III and I disorders according to RDC/TMD were included in their study group. Like our study smoking patients were not included in the study as it would increase their OS level. As a result of their study, they found high levels of both 8-OHdG and MDA in patients with higher scores of pain intensity and decreased concentrations of total antioxidant status (TAS) were associated with pain intensity. In a similar study, Vrbanovic et al. [8] investigated salivary OS markers (glutathione peroxidase, superoxide dismutase, total antioxidant capacity (TAC), uric acid, 8-OHdG, MDA) and salivary cortisol (SC) as a stress indicator, between 20 TMD patients and 15 healthy control subjects. Their inclusion criteria were painful DD or myofascial pain (MP) according to the Diagnostic Criteria of TMD (DC/TMD). In contrast, they found significantly higher levels of TAC in TMD patients in comparison with controls. Lawaf et al. [11] compared saliva and serum samples from 28 patients with painful TMD, 28 patients with painless TMD, and 28 healthy controls. They found a significant relationship between TMD and pain and serum TAC levels, but no significant relationship was found between salivary TAC levels and TMD. Almeida et al. [32] investigated salivary Total OS (TOS) level and TAC of 30 patients with painful TMD and 30 healthy controls. As a result of the study, they found a significant relationship between salivary TAC level and OS index and painful TMD, but no significant relationship was found between TOS level and painful TMD. No correlation was found between the TAC level and TOS level and VAS values of the patients. Kobayashi et al. [33] compared anxiety levels and salivary stress biomarkers alpha-amylase and cortisol levels in children with and without TMD. Although stress scores of children were significantly higher in the TMD group, no significant difference was found between the groups in terms of salivary stress biomarkers. Richards et al. evaluated blood OS markers in individuals with TMD symptoms as well as chronic muscle pain and observed an association between increased OS levels and total free radicals [20]. However, the patients included in the study were patients with chronic fatigue syndrome, who are self-reporting joint dysfunction and muscle pain symptoms.

The difference in our study from other studies is that our study did not specifically evaluate painful TMD patients. We found no difference between the study and control group according to saliva and blood OS and inflammation marker levels. However, when VAS values were evaluated, a strong positive correlation was observed only in serum IL-6 and 8-OHdG levels. Although there was no difference between the study and control groups in our study, the strong association between pain and IL-6 and 8-OHdG levels suggests that these mediators would be particularly useful in the assessment of pain-related TMD. In our study, the study group was composed of RDC/TMD Group 2 patients. This group is subdivided into three groups: DD with reduction, DD without reduction-with, and without limited opening. We believe that conducting studies with specific subgroups may lead to different results. Since the primary goal in the treatment of these patients is pain relief and restoration of function, these results also indicate the significant role of antiinflammatory treatment in therapy.

There are several studies in the literature investigating the OS and inflammation markers in TMJ, but it is noticeable that different results are obtained for the different markers. One reason for the variability in those study results may be the examination of different mediators in each study. Due to the significant differences in both the patient groups and the mediators studied in various studies, a conclusive result cannot be reached. Considering the existing controversies in this respect in the literature, this study sought to assess the serum and saliva IL-6, 8-OHdG, and MDA of TMD patients and make a comparison with healthy subjects. No significant difference was found between the groups. This could be because these mediators may not play a direct role in the pathogenesis of the disease or may not be present in quantities distinctive enough in blood and saliva. However, the strong association of these mediators with pain in our study may suggest a potential role of inflammation and OS in the pathogenesis of the disease. This diversity in results may also be associated with the fact that OS is under the influence of many factors such as age, sex, cultural-psychosocial status, and dietary habits. Creating more specific study groups by eliminating the potential effective factors (e.g., by reducing the age range, homogenizing sex, or standardizing groups according to dietary habits, etc.) would increase the reliability of the results obtained.

The main limitation of this study is also the high variability of OS markers. OS mediators can vary significantly from person to person and are very difficult to standardize in human scientific studies. In our study, similar to other studies in the literature, parameters such as smoking or alcohol use were excluded from the study, and the age range was evaluated within itself. Aging is a potential risk factor for many chronic diseases. As the antioxidant capacity decreases with aging in living organisms, oxidative damage increases [34]. In our study, the mean ages of the study and control groups were kept close to each other so that age-related oxidative damage would not affect the results. When all healthy and TMD patients were evaluated, no correlation was found between age and serum and saliva 8-OHdG, MDA, and IL-6 levels. This result is similar to that of Sotillo et al. [5]. When the patients with joint disease were divided into two groups as under 18 years of age and over, no difference was found between the serum and saliva 8-OHdG, MDA, and IL-6 levels between the two groups. Some studies also standardize sex in free radical measurements. Sotillo et al. used only female samples in their study to eliminate the effect of sex-related hormonal changes between the study and control groups [5]. Lawaf et al., on the other hand, stated that the TAC was not related to age and sex as a result of their studies [11]. In our study, the control and study groups were formed without discriminating between men and women.

Literature reveals that free radicals and inflammatory mediators are higher in the synovial fluid of TMD patients [24,28,31]. Since the synovial membrane is semipermeable, it is stated that these mediators can pass into the systemic circulation, albeit at a low level [34,35]. However, these mediators are already present at a certain level in the systemic circulation and this level is highly affected by environmental factors. Therefore, synovial fluid analysis, despite being a more invasive method, may give more accurate results for assessing the levels of OS and inflammation markers in TMD patients, as it may be less affected by environmental factors.

In conclusion, we found no significant relationship by means of inflammatory and OS markers between the TMD patients and healthy groups. However, strong inter-group correlation supports the hypothesis that pain, inflammation, and OS mechanisms are related to each other in TMD patients. This relationship was not detected in saliva samples. Further studies are needed with more specific TMD subtypes to better understand the molecular etiopathogenesis of the disease.

## Figures and Tables

**Table 1:** Demographic data of the study population.

	Age	Sex (Female/Male)
**Control group** n = 17	24.82 ± 7.04	6/11
**Study group** n = 27	26.51 ± 14	24/3
**Total** n = 44	25.86 ± 11.73	30/14

**Table 2:** Comparison of gender with serum and saliva 8-OHdG, IL-6, and MDA levels. No statistically significant difference between serum and saliva 8-OHdG, IL-6- and MDA levels of male and female subjects. (8-OHdG: 8-hydroxydeoxyguanosine (ng/mL), IL-6: Interleukin-6 (ng/mL), MDA: Malondialdehyde (μm)).

	Sex	Median (min-max)	p
**(8-OHdG) Serum**	Female	19.7 (7.8–89.3)	0.313
	Male	28.1 (12.3–79.1)	
**(8-OHdG) Saliva**	Female	14.5 (3–39.6)	0.918
	Male	15.3 (2.6–95.1)	
**IL-6 Serum**	Female	125.5 (56.5–363.6)	0.73
	Male	112.4 (69.6–411)	
**IL-6 Saliva**	Female	62.3 (5.1–443.7)	0.677
	Male	49.5 (5.8–183.3)	
**MDA Serum**	Female	0.1 (0.1–0.6)	0.266
	Male	0.1 (0.1–0.7)	
**MDA Saliva**	Female	0.3 (0.1–1.4)	0.65
	Male	0.2 (0.01–0.8)	

**Table 3:** Serum and saliva 8-OHdG, IL-6 and MDA median values. No significant difference between the groups in terms of 8-OHdG, IL-6, and MDA levels. (8-OHdG: 8-hydroxydeoxyguanosine (ng/mL), IL-6: Interleukin-6 (ng/mL), MDA: Malondialdehyde (μm))

		Median (min-max)	Mean rank	Test statistic	p
**(8-OHdG) Serum**	Control	29.7 (11–71.1)	29.7	U = 116	0.358
	TMD	19.7 (7.8–89.3)	20		
**(8-OHdG) Saliva**	Control	14 (2.6–45.3)	14	U = 150	0.301
	TMD	16.9 (3.3–95.1)	17		
**IL-6 Serum**	Control	113 (56.5–411)	113	U = 168	0.951
	TMD	123.8 (58–397.8)	124		
**IL-6 Saliva**	Control	40.2 (5.8–443.7)	40.2	U = 221	0.838
	TMD	51.8 (0–154.8)	52		
**MDA Serum**	Control	0.1 (0.1–0.7)	0.1	U = 136	0.307
	TMD	0.1 (0.1–0.4)	0.1		
**MDA Saliva**	Control	0.2 (0–1.4)	0.2	U = 197	0.433
	TMD	0.2 (0–1.4)	0.2		

**Table 4:** A strong positive correlation was found between pain and serum 8-OHdG and IL-6 levels. Spearman’s rho (8-OHdG: 8-hydroxydeoxyguanosine, IL-6: Interleukin-6, MDA: Malondialdehyde).

	(8-OHdG) Serum	(8-OHdG) Saliva	IL-6 Serum	IL-6 Saliva	MDA Serum	MDA Saliva
**VAS**	r = 0.604	r = 0.411	r = 0.713	r = 0.171	r = −0.022	r = −0.26
